# *Neisseria gonorrhoeae* ciprofloxacin susceptibility testing and *gyrA* targets

**DOI:** 10.1016/S2666-5247(23)00150-7

**Published:** 2023-05-23

**Authors:** Daniel H F Rubin, Tatum D Mortimer, Yonatan H Grad

**Affiliations:** Department of Immunology and Infectious Diseases, Harvard TH Chan School of Public Health, Boston, MA, USA (DHFR, TDM, YHG)

Authors’ reply

We agree with Jacob A Tickner and colleagues that diagnostics that predict *Neisseria gonorrhoeae* susceptibility to ciprofloxacin based on *gyrA* codon 91 are valuable tools in the effort to treat gonorrhoea and to address the challenge of antimicrobial resistance. But will these diagnostic tests always have the sensitivity and specificity that they have today? The premise of our work is that a diagnostic that guides antibiotic use will itself apply a selective pressure to escape from that diagnostic, under the same principle as antibiotics selecting for resistance.^1^ Our in vitro results suggest that diagnostic escape is plausible. Moreover, this process promotes a pathway to ciprofloxacin resistance that also confers resistance to zoliflodacin, a novel antibiotic^2^ in phase 3 trials. With *gyrA* codon 91-based diagnostics only recently coming into use, we are just at the start of the natural experiment to see what will happen in vivo. The more widespread the uptake of these diagnostics, the greater the selective pressure.

When do potential adverse outcomes merit preventative action? Answering this question requires balancing the costs and benefits of action and inaction and decision making under uncertainty. We agree with Tickner and colleagues that it is important to weigh the likelihood of the outcome and to enumerate the costs and benefits—for example, the cost of adding a locus to a diagnostic test and the cost of waiting until the detection of clinical isolates with diagnostic escape mutations—to arrive at a reasoned strategy.

We suggest that there is a clear need for vigilant monitoring for the emergence of diagnostic escape mutations through evaluation of treatment failures alongside expanded surveillance with phenotyping and genome sequencing of clinical isolates.^3,4^ Surveillance for these mutations might be even more relevant with the rollout of zoliflodacin, as selection for resistance-conferring mutations in *gyrB* might also facilitate escape from *gyrA* codon 91-based diagnostics.
